# Gait Assessment Using Three-Dimensional Acceleration of the Trunk in Idiopathic Normal Pressure Hydrocephalus

**DOI:** 10.3389/fnagi.2021.653964

**Published:** 2021-03-10

**Authors:** Shigeki Yamada, Yukihiko Aoyagi, Masatsune Ishikawa, Makoto Yamaguchi, Kazuo Yamamoto, Kazuhiko Nozaki

**Affiliations:** ^1^Department of Neurosurgery, Shiga University of Medical Science, Shiga, Japan; ^2^Interfaculty Initiative in Information Studies/Institute of Industrial Science, The University of Tokyo, Tokyo, Japan; ^3^Department of Neurosurgery and Normal Pressure Hydrocephalus Center, Rakuwakai Otowa Hospital, Kyoto, Japan; ^4^Digital Standard Co., Ltd., Osaka, Japan; ^5^Rakuwa Villa Ilios, Rakuwakai Healthcare System, Kyoto, Japan

**Keywords:** idiopathic normal pressure hydrocephalus, acceleration sensor, pathological gait, gait assessment, trunk acceleration, gait analysis, smartphone device

## Abstract

**Background:** The subjective evaluation of pathological gait exhibits a low inter-rater reliability. Therefore, we developed a three-dimensional acceleration of the trunk during walking to assess the pathological gait quantitatively.

**Methods:** We evaluated 97 patients who underwent the cerebrospinal tap test and were diagnosed with idiopathic normal pressure hydrocephalus (iNPH) and 68 healthy elderlies. The gait features of all patients were evaluated and classified as one of the following: freezing of gait, wide-based gait, short-stepped gait, shuffling gait, instability, gait festination, difficulty in changing direction, and balance disorder in standing up. All gait features of 68 healthy elderlies were treated as normal. Trunk acceleration was recorded automatically by a smartphone placed on the umbilicus during a 15-foot walking test. Two novel indices were created. The first index was a trunk acceleration index, which was defined as (forward acceleration fluctuation) + (vertical acceleration fluctuation) – (lateral acceleration fluctuation) based on the multivariate logistics regression model, and the second index was created by multiplying the forward acceleration with the vertical acceleration. Additionally, 95% confidence ellipsoid volume of the three-dimensional accelerations was assessed.

**Results:** Forward and vertical acceleration fluctuations were significantly associated with the probability of an iNPH-specific pathological gait. The trunk acceleration index demonstrated the strongest association with the probability of an iNPH-specific pathological gait. The areas under the receiver-operating characteristic curves for detecting 100% probability of an iNPH-specific pathological gait were 86.9% for forward acceleration fluctuation, 88.0% for vertical acceleration fluctuation, 82.8% for lateral acceleration fluctuation, 89.0% for trunk acceleration index, 88.8% for forward × vertical acceleration fluctuation, and 87.8% for 95% confidence ellipsoid volume of the three-dimensional accelerations.

**Conclusions:** The probability of a pathological gait specific to iNPH is high at the trunk acceleration fluctuation, reduced in the forward and vertical directions, and increased in the lateral direction.

## Introduction

Gait and balance impairments are the predominant symptoms of idiopathic normal pressure hydrocephalus (iNPH). The pathological gait that is specific to iNPH has been characterized as the freezing of gait, wide-based gait, short-stepped gait, shuffling gait, instability, gait festination, difficulty in changing direction, and balance disorder in standing up (Stolze et al., 2000, 2001; Marmarou et al., 2005; Ishikawa et al., 2019a). Video-recorded gait performance before and after the spinal tap test and shunt surgery has been recommended for gait assessments of patients with iNPH (Marmarou et al., 2005; Mori et al., 2012; Ishikawa et al., 2019a; Nakajima et al., 2021). However, pathological gait is generally evaluated subjectively and exhibits no standardized rating system. We previously investigated the inter-rater reliability on the gait features of patients with iNPH by viewing videos of the timed-up-and-go test (TUG) in blinded fashion (Ishikawa et al., 2019a). As a result, the agreement on gait features among multiple raters was lower than expected, even among doctors or physiotherapists who are sufficiently experienced to diagnose patients with iNPH. Gait disturbance in iNPH has been assessed objectively by various quantitative measurement instruments. For example, a reduced stride length and diminished step height are typical spatiotemporal and kinematic characteristics of gait in iNPH (Stolze et al., 2000; Williams et al., 2008; Agostini et al., 2015; Schniepp et al., 2016; Yang et al., 2016; Kitade et al., 2018; Ferrari et al., 2020). However, these quantitative assessments have not been used to evaluate pathological gait in iNPH. Additionally, measurement instruments, such as three-dimensional (3D) optical motion capture or force plates, are not been commonly used in general practice, as they are expensive and heavy, and therefore are difficult to carry, which increases the preparation time for measurement. Therefore, to overcome these limitations with a device that is practical for clinical research or practice, we analyzed gait using a smartphone equipped with high-precision inertial sensors that detect changes in tilt, rotation, and acceleration in three dimensions, as well as a free application (SENIOR Quality, Digital Standard Co., Ltd.) to assess walking biomechanics (Ishikawa et al., 2019b; Yamada et al., 2019). In our previous study, we found that time on TUG and chronological fluctuations of 3D acceleration of the trunk during TUG are important markers for evaluating the severity of gait disturbance in iNPH (Yamada et al., 2019). However, we had not assessed the pathological gait specific to iNPH using smartphone inertial sensors.

The trunk acceleration fluctuations were reported to be a useful measure to identify frail and balance dysfunction, compared to the stride and stride-to-stride fluctuations (Moe-Nilssen and Helbostad, 2005). Whether the pathological gait specific to iNPH is associated with the trunk acceleration fluctuations and which directions of them are closely associated to the iNPH-specific gait are still unclear. Therefore, the present study aimed to assess the potential relationships between the pathological gait specific to iNPH and 3D trunk acceleration fluctuations during simple, straight walking.

## Materials and Methods

### Study Population

This observational study was approved by the local institutional ethics committee (Raku-Oto-Rin-17-010). Details of the inclusion criteria, characteristics of the study population, image acquisition, and methods of data collection were described previously (Ishikawa et al., 2019a,b; Yamada et al., 2019). From March 2017 to September 2019, 134 patients who were suspected of presenting with iNPH underwent a cerebral spinal fluid (CSF) tap test, which consisted of removing ≥30 mL CSF via a lumbar tap to evaluate the response. Patients diagnosed with secondary normal pressure hydrocephalus (sNPH) that developed after subarachnoid hemorrhage or trauma were excluded from this study. After evaluation, 97 patients who met the inclusion criteria and whose 3D trunk acceleration data during simple, straight walking were successfully obtained were included in this study. All patients or their representatives gave written informed consent, and their private information was anonymized in a linkable manner at each institute. Improvements of gait and cognitive symptoms were assessed by the iNPH grading scale (Ishikawa, 2004; Mori et al., 2012; Nakajima et al., 2021), with quantitative examinations given before, 1 day, and 4 days after the CSF tap test. All patients underwent brain and whole-spine MRI and single-photon emission computed tomography to determine in the differential diagnosis or coexistence of Alzheimer's disease, cerebral infarction, and cervical or lumber canal stenosis, etc. Coexistence of Alzheimer's disease was diagnosed based on their behavioral and psychological symptoms of dementia, cognitive function tests, several imaging scans, and phosphorylated tau in CSF. However, in this study, we examined the relationship between the assessment of pathological gait and 3D acceleration of the trunk without considering diagnoses of definitive iNPH and co-morbidities. In addition, 68 elderly individuals aged 60 and over who were using the rehabilitation facility for day care services and who did not have gait disturbance were included as a control. They agreed to participate in this study and measured the 3D trunk acceleration using the SENIOR Quality app.

### Gait Assessment

A subjective assessment of pathological gait specific to iNPH was performed as the freezing of gait (which was referred to as a magnetic foot response or brief arrest in which the feet appeared to be stuck to the floor, especially upon start, turn, or changing direction), wide-based gait, short-steps (or senile) gait (reduced stride length), shuffling gait (diminished step height), instability (unsteady gait), gait festination, difficulty in changing direction, and balance disorder in standing up, which was assessed on a 3-point grading scale (0 = none; 1 = likely positive; 2 = positive) based on video records of TUG performed twice as described in our previous study (Ishikawa et al., 2019a). The gait features of 97 patients were assessed by the attending doctors and physiotherapists before the tap test. Because each pathological gait was related to the others and to the severity of gait disturbance, we created a new parameter that indicates a pathological gait specific to iNPH, which is calculated as (the sum of each evaluation point of eight pathological gait features)/16 × 100 (%). Compared to each assessment of pathological gait specific to iNPH, the probability of iNPH-specific gait was reliable, because it was significantly associated with the gait domain of the Japanese iNPH grading scale which was rated as normal, complaints of instability, walks without supportive devices, walks with supportive devices, and unable to walk (Ishikawa, 2004; Mori et al., 2012; Nakajima et al., 2021). All gait features of 68 healthy elderlies were treated as normal, i.e., the probability of iNPH-specific gait was rated as 0%.

### Data Analysis

**Figure 1:**
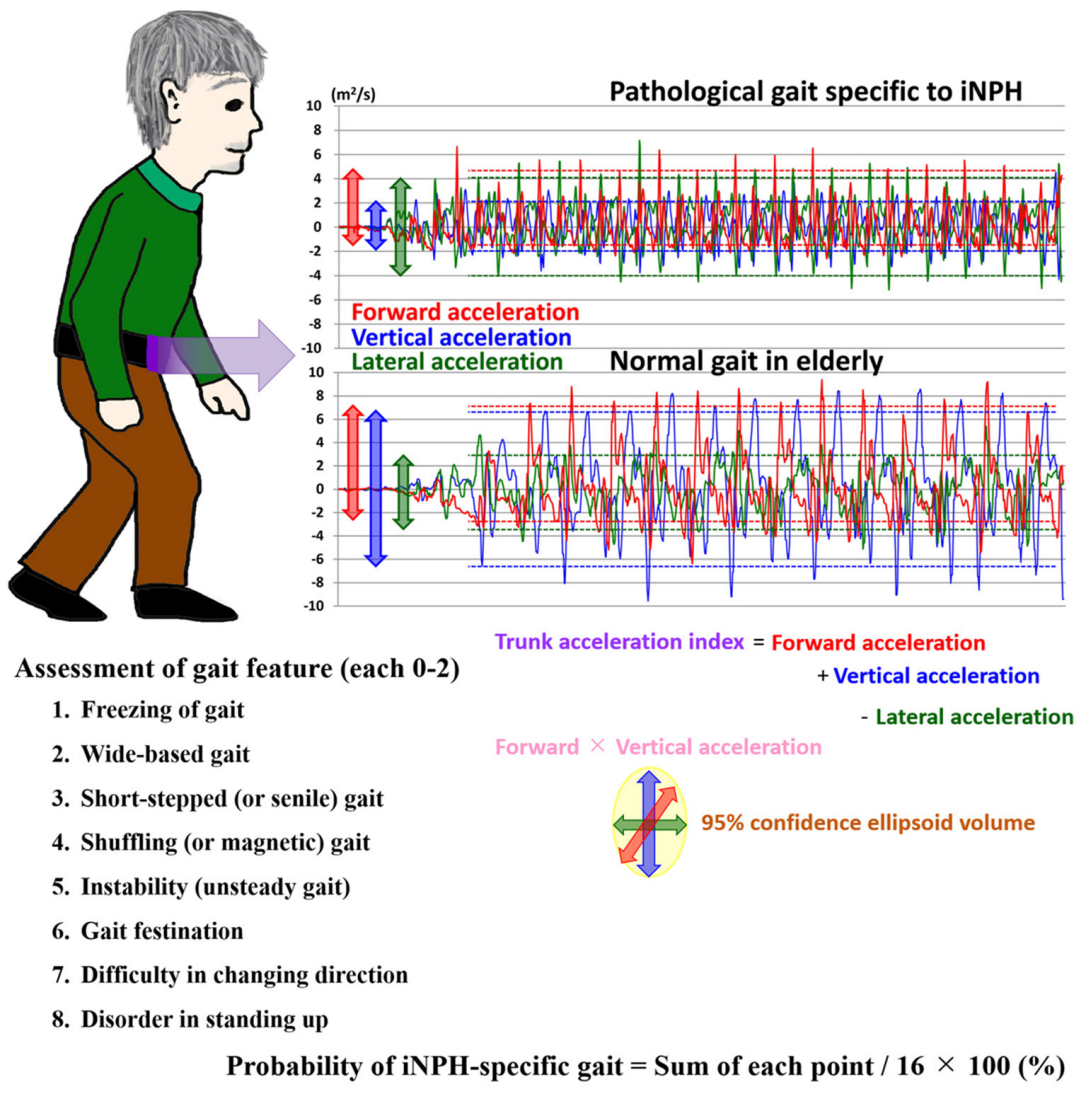
Trunk accelerations in three axial directions during straight walking and the probability of an iNPH-specific pathological gait. Graphs show chronological changes in acceleration every 0.01 s in three axial directions during a 15-foot walking test by an accelerometer application on an iPhone. The upper graph depicts measurements of a representative patient with iNPH, and the lower graph depicts measurements of a healthy elderly volunteer. The red line indicates the forward acceleration forward (>0) and backward (<0); blue indicates the vertical acceleration upward (>0) and downward (<0); and green indicates the lateral acceleration toward the left (>0) and right (<0). The dotted lines indicate the 95% confidential intervals of acceleration amplitudes.

All patients completed a 15-foot straight walking test using a free iPhone application (SENIOR Quality). Patients placed the iPhone in a small pouch on their umbilicus. Starting in the standing position, they walked straight for more than 10 m at their preferred walking speed. The application stopped recording automatically when patients reached their 15th step. By using an inertial accelerometer and gyroscope in the iPhone, acceleration and angular speed in three axial directions were automatically recorded every 0.01 s and then automatically stored on the cloud server (Microsoft Azure; Microsoft Corporation, Redmond, WA, USA). After a comprehensive parameter search, we found a significant relationship between 3D trunk accelerations and the pathological gait. The 3D trunk accelerations changed periodically during the 15-foot straight walking test, but their amplitudes were different in each step, as shown in Figure 1. Therefore, fluctuations in the 3D acceleration were defined as a 95% confidence interval (CIs) of the amplitude of the acceleration in each direction. In the first analysis, even the most promising parameters were insufficient for a reliable indicator of an iNPH-specific pathogenic gait. Therefore, we investigated possible combinations of several parameters that could detect the pathological gait more reliably. After a comprehensive parameter search, we selected two candidates that could be used to detect a pathological gait specific to iNPH. The first was defined as the trunk acceleration index, the formula for which was defined as (forward acceleration fluctuation) + (vertical acceleration fluctuation) – (lateral acceleration fluctuation) based on the following multivariate logistics regression model calculation; probability of iNPH-specific gait = {−0.96 × (forward acceleration fluctuation) + −1.29 × (vertical acceleration fluctuation) + 0.10 × (lateral acceleration fluctuation)} × 100. The second was defined as simply multiplying the forward acceleration and the vertical acceleration. In addition, we assessed the volume of the 95% confidence ellipsoid (95% CE) for 3D plots of chronological changes of tri-axial accelerations, which were calculated as 4π/3 × (maximum length of the axis from the center to the ellipse) × (minimum length) × (length of the axis orthogonal to the two axes), as described in our previous report (Yamada et al., 2019).

### Statistical Analysis

Continuous variables were compared using the Wilcoxon rank-sum test, and proportions of variables were compared using Fisher's exact test in two groups. The relationships between the parameters and the probability of an iNPH-specific gait index were compared using Pearson's correlation coefficient (*r*). Additionally, the area under the receiver-operating characteristic curves (AUCs) for detecting the probability of a pathological gait specific to iNPH by 3D trunk acceleration fluctuations and the combined parameters were calculated to evaluate the optimal thresholds for maximizing the sum of sensitivities and specificities. Based on the distribution of the probability of a pathological gait specific to iNPH, 70 and 100% of the probability were adapted as two threshold values. Missing data were treated as deficit data that did not affect other variables. *P*-values < 0.05 were considered to be statistically significant. Statistical analyses were performed using R software (version 4.0.3. R Foundation for Statistical Computing, Vienna, Austria. http://www.R-project.org).

## Results

### Clinical Characteristics

**Figure 2:**
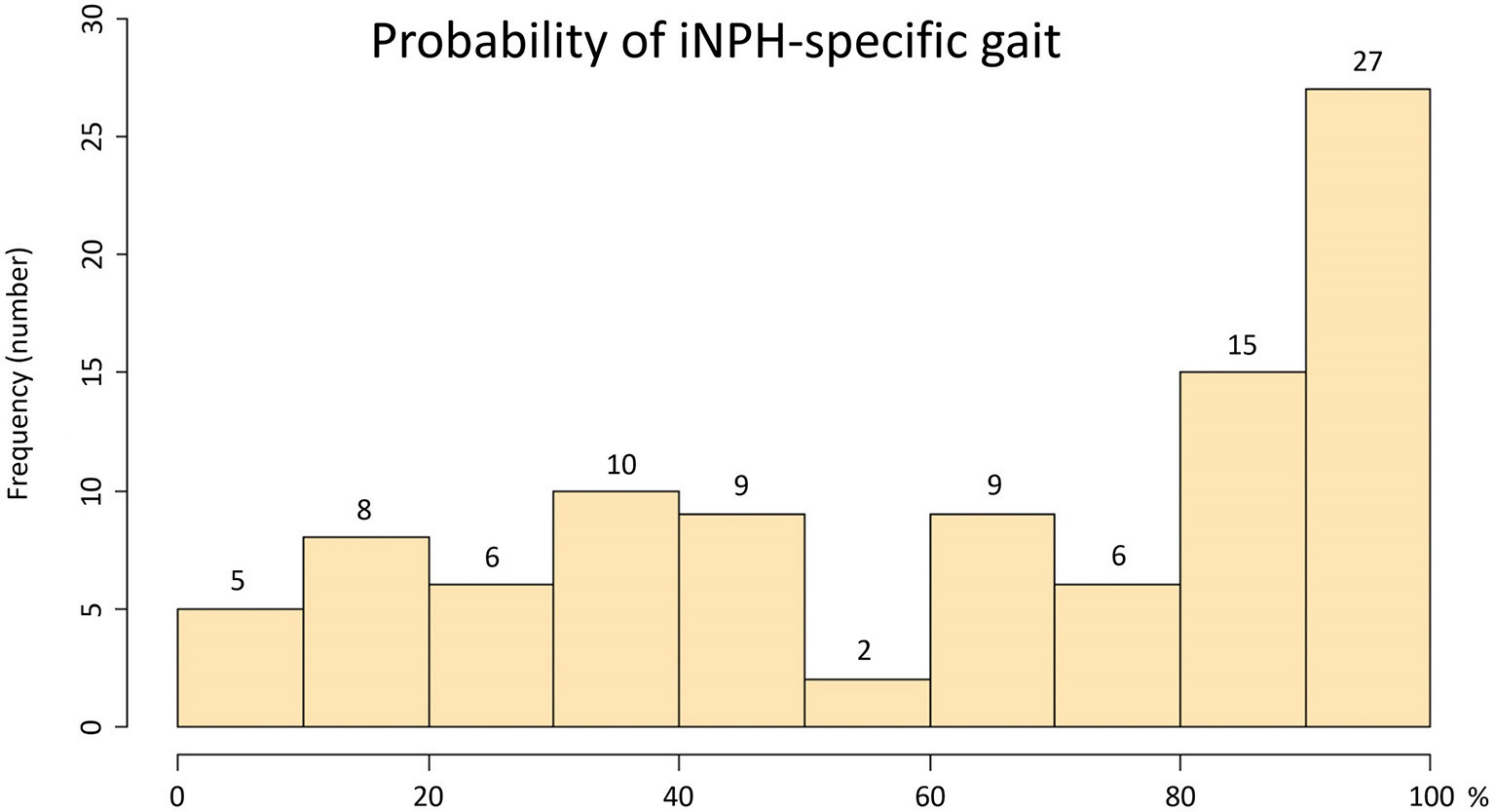
Histogram of the probability of an iNPH-specific pathological gait. The x-axis shows the probability of an iNPH-specific pathological gait (%), and the y-axis shows the frequency. The probability of an iNPH-specific pathological gaitwas the sum of each point (max: 2 points) of following eight gait features/16 × 100 (%); Gait feature: 1. freezing of gait, 2. wide-based gait, 3. short-steps gait, 4. shuffling gait, 5. instability, 6. gait festination, 7. difficulty in changing direction, 8. balance disorder in standing up.

Ninety-seven patients (mean age, 76.9 ± 7.3 years; 63 males, 34 females) met our inclusion criteria. Their clinical characteristics are shown in Table 1. Based on the response to the CSF tap test, 84 patients were diagnosed with possible iNPH, and 13 patients were judged as negative to the tap test. After that, 56 of 84 patients with a positive response to the tap test and 2 of 13 patients without a response underwent a ventriculo-peritoneal shunt surgery. Because all patients exhibited some improvement in their symptoms after shunt surgery, they were diagnosed with definite iNPH, and two of them were determined to present with a false negative response to the tap test. The coexistence rate of spinal disease (cervical or lumber canal stenosis) in the tap-negative group was significantly higher than that of the tap-positive group (Table 1). Seventy-five patients (77%) presented with a history of falls, and surprisingly, more than half presented with a history of three or more falls. The percentage of severe grade on the modified Rankin scale and on the gait domain of the iNPH grading scale was significantly higher for the tap-positive group than for the tap-negative group. Wide-based gait, short-steps gait, shuffling gait, instability, gait festination, difficulty in changing direction, and balance disorder in standing up were observed in more than half of the patients. The tap-positive group was significantly more likely to exhibit the short-steps gait and difficulty in changing direction than the tap-negative group. Figure 2 shows the frequency of the probability of the pathological gait specific to iNPH, which was based on an integrated scale of eight pathological gait features in the 97 patients. The median and quartile range of the probability of an iNPH-specific gait were 64% and 31–100%. No patients without any pathological gait features were present. The probability of an iNPH-specific gait was significantly associated with the modified Rankin scale (*r* = 0.59, 95% CI = 0.45–0.71) and the gait domain of the iNPH grading scale (0.71, 0.59–0.80). In addition, 68 participants (mean age, 79.1 ± 6.6 years; range, 62–92 years; 32 males, 36 females) were included as age-adjusted healthy controls.

**Table 1:** Clinical characteristics in this study.

	**Total**	**Tap-positive**	**Tap-negative**	***P*-value**
Total number	97	84	13	
Mean (± SD) age, years	76.9 ± 7.3	76.4 ± 7.5	80.2 ± 5.4	0.067
CSF Shunt surgery	58 (60%)	56 (67%)	2 (15%)	**<0.001**
Co-morbidity				
Alzheimer's disease	39 (40%)	33 (39%)	6 (46%)	0.763
Spinal disease	28 (29%)	21 (25%)	7 (54%)	**0.048**
Stroke	5 (5%)	4 (5%)	1 (8%)	0.521
Male: Female	63: 34	55: 29	8: 5	0.765
History of falls				
none: 1 or 2 times: ≥3 times	22: 14: 61	17: 14: 53	5: 0: 8	0.149
modified Rankin scale				
0 or 1: 2: 3: 4: 5	0: 48: 38: 9: 2	0: 39: 37: 6: 2	0: 9: 1: 3: 0	**0.026**
Severity of gait on iNPHGS				
1: 2: 3: 4	8: 43: 36: 10	7: 36: 35: 6	1: 7: 1: 4	**0.016**
Gait feature (none: likely positive: positive)				
Freezing of gait	53: 7: 37	44: 7: 33	9: 0: 4	0.571
Wide-based gait	11: 23: 63	9: 18: 57	2: 5: 6	0.198
Short-stepped gait	15: 16: 66	14: 9: 61	1: 7: 5	**0.002**
Shuffling gait	18: 18: 61	14: 14: 56	4: 4: 5	0.112
Instability	7: 28: 62	5: 23: 56	2: 5: 6	0.181
Gait festination	42: 14: 41	33: 14: 37	9: 0: 4	0.096
Difficulty in changing direction	17: 23: 57	11: 21: 52	6: 2: 5	**0.023**
Difficulty in standing	47: 12: 38	40: 10: 34	7: 2: 4	0.766
Probability of iNPH-specific gait (%)	64.0 ± 32.2	66.2 ± 31.6	49.5 ± 33.8	0.120

*Values in bold are statistically significant, i.e. P values < 0.05*.

### Relationship Between the Pathological Gait and Acceleration Fluctuations During Walking

**Figure 3:**
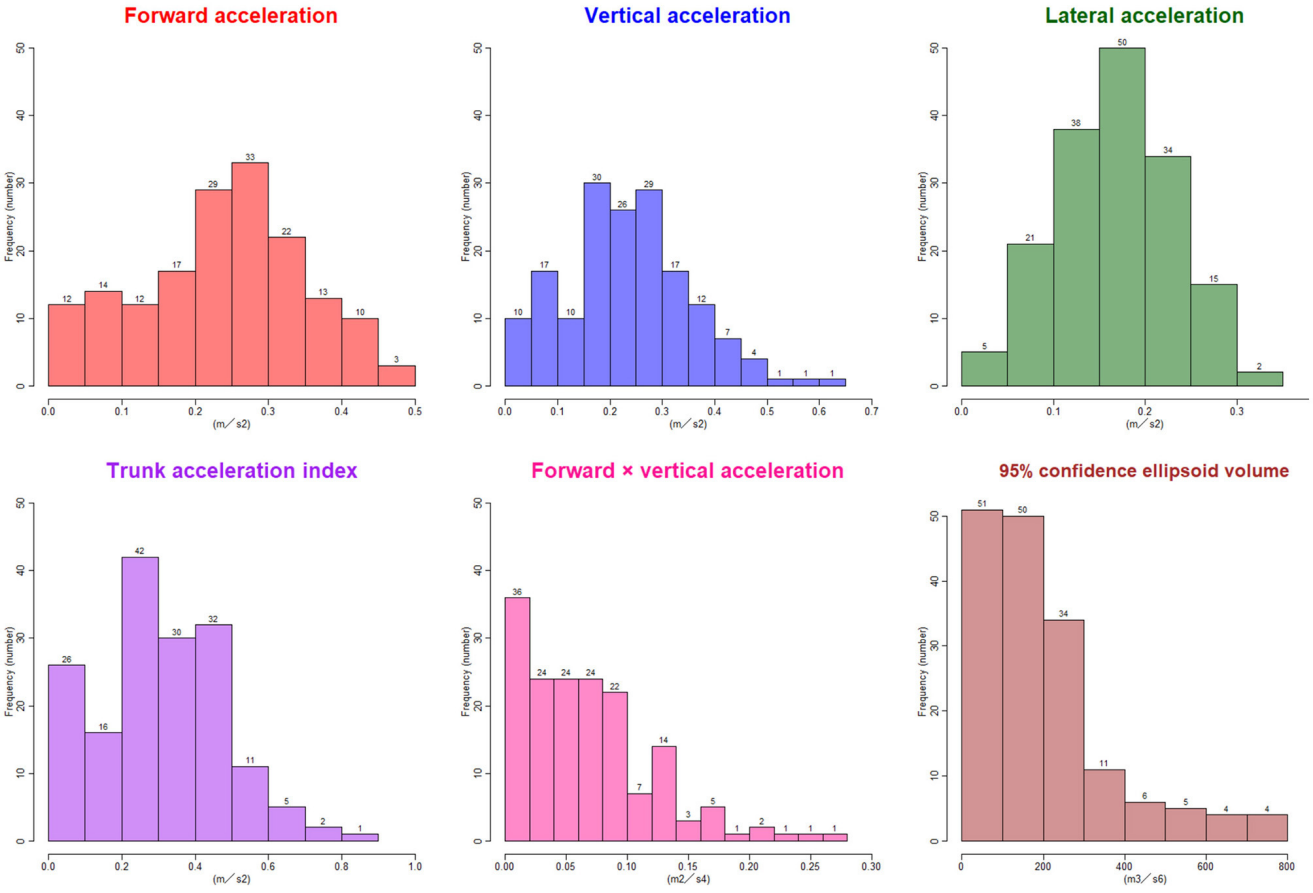
Histograms of the trunk acceleration fluctuations in each three axial direction, trunk acceleration index, forward × vertical acceleration fluctuation, and 95% confidence ellipsoid volume. The x-axes show the distribution of the parameters, and the y-axes show the frequencies.

**Figure 4:**
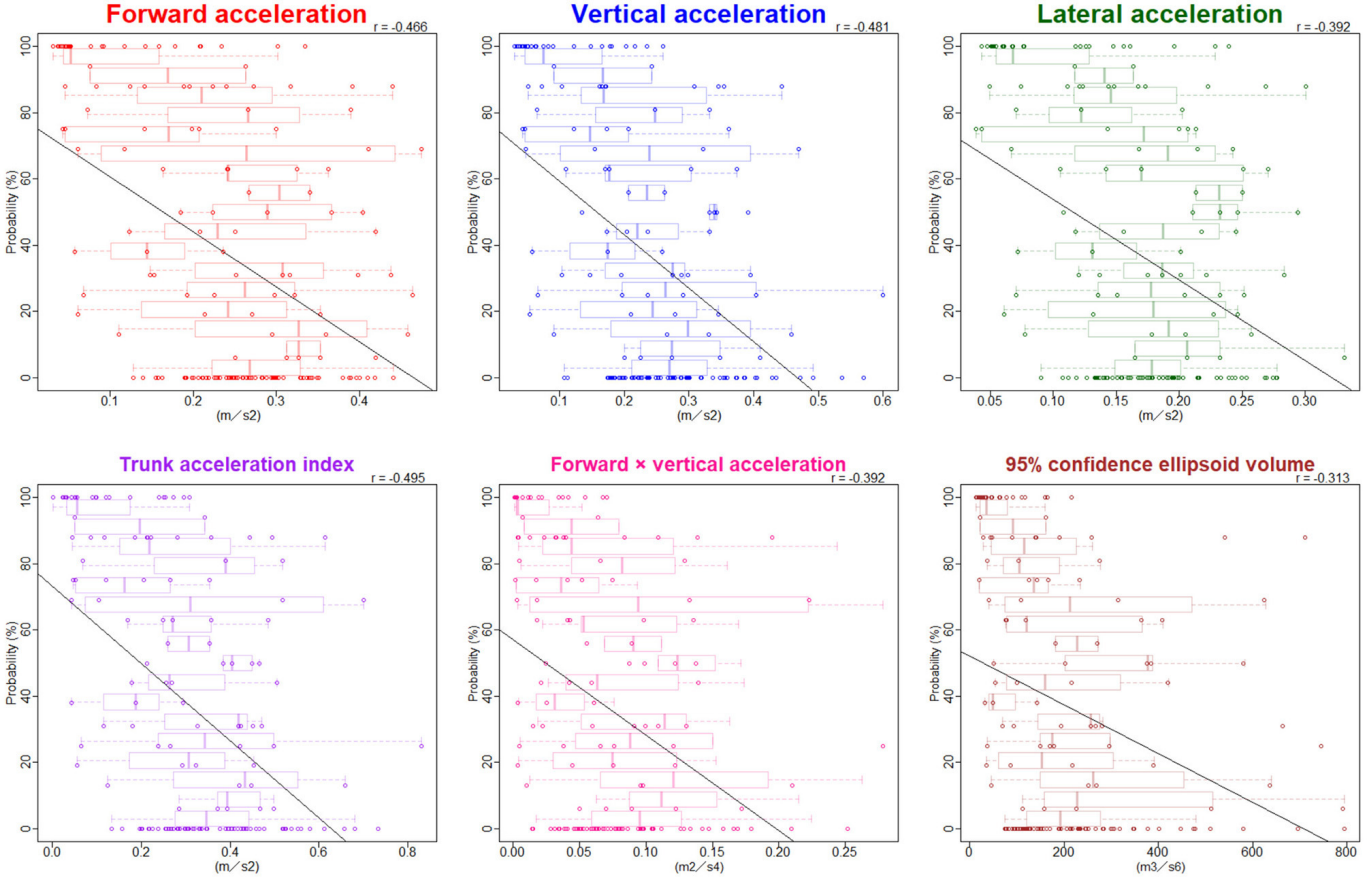
Relationship between the probability of an iNPH-specific pathological gait and trunk acceleration fluctuations. Combination graphs of scatter plots and box plots show the relationships between the probability of an iNPH-specific pathological gait and trunk acceleration fluctuations in each three direction and combined directions. The black lines indicate regression lines. Pearson's correlation coefficient (*r*) is shown in the upper right of each graph.

The ranges of trunk acceleration fluctuations in the forward, vertical, and lateral directions were 0.03–0.47, 0.03–0.60, 0.04–0.33 m/s^2^, respectively. As shown in Figure 3, the distribution of the trunk acceleration index was similar to that of vertical acceleration fluctuation, whereas that of the forward × vertical acceleration fluctuation was similar to that of 95% CE volume of 3D acceleration. Of the three directions, the most significant association with the probability of an iNPH-specific pathological gait was the vertical trunk acceleration fluctuation (*r* = −0.48) and the next was forward acceleration fluctuation (−0.47), as shown in Figure 4. The trunk acceleration index exhibited the strongest association with the probability of an iNPH-specific pathological gait (−0.50), whereas the forward × vertical acceleration fluctuation (−0.39) and 95% CE volume exhibited a lower association (−0.31) compared to the trunk acceleration fluctuations in three directions.

**Figure 5:**
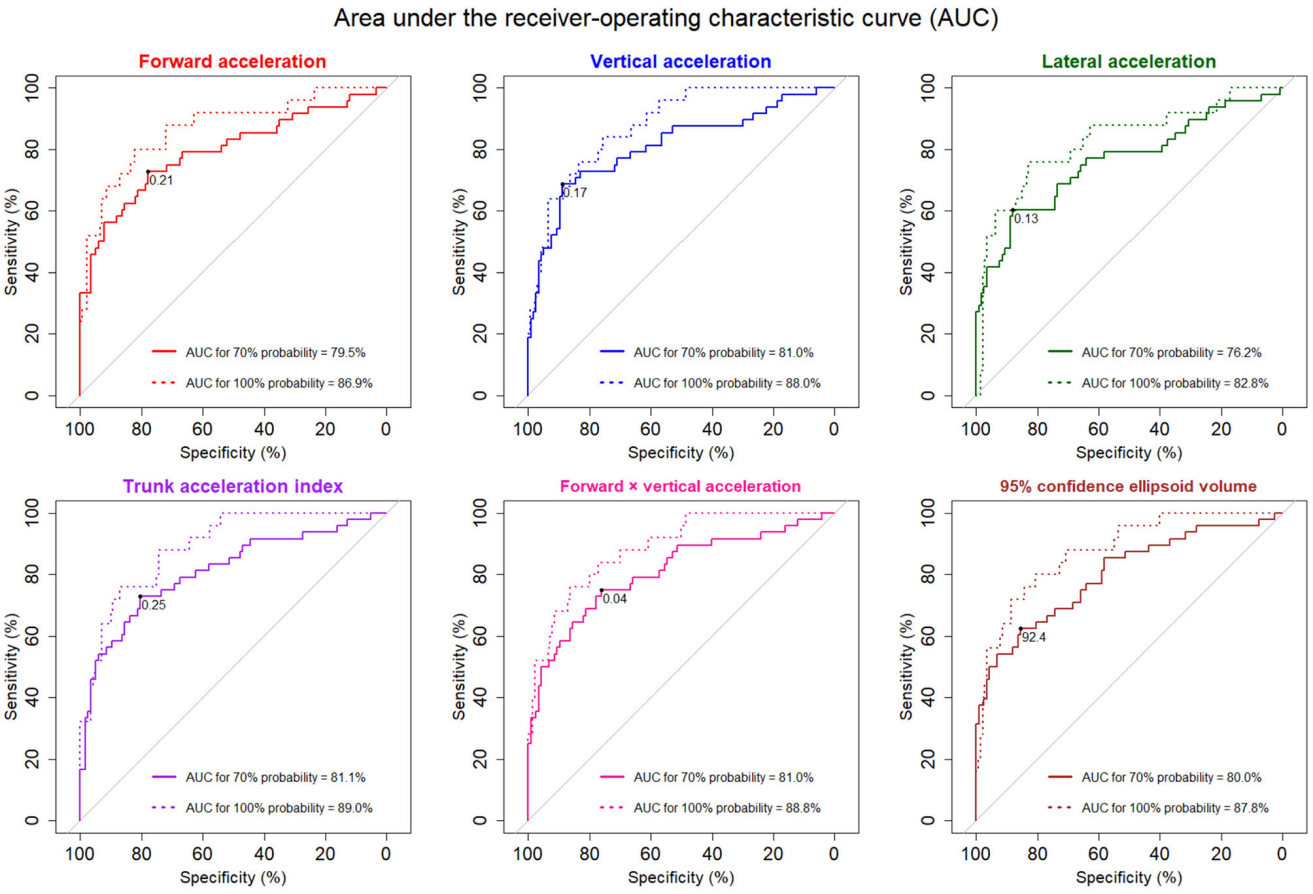
The receiver-operating characteristic (ROC) curves for detecting pathological gait specific to iNPH. The solid ROC curves indicate 70% probability of pathological iNPH-specific gait, and the dotted curves indicate 100% probability. The areas under the ROC curves (AUCs) for 70 and 100% possibilities of pathological iNPH-specific gait were added to each graph. The optimal thresholds for detecting 70% probability of a pathological iNPH-specific gait are marked at the black points.

The AUCs for detecting 70% probability of an iNPH-specific pathological gait were 76–81% in the 3D acceleration fluctuations and the combined parameters, whereas the AUCs for detecting 100% probability were more than 86% for all directions and combinations of parameters except for the lateral acceleration fluctuation (Figure 5).

## Discussion

Using an inertial accelerometer built into an iPhone and a free application (SENIOR Quality), we attempted to assess the pathological gait specific to iNPH based on trunk acceleration fluctuations in three axial directions during simple, straight walking across a short distance. Of the trunk acceleration fluctuations in three directions, forward and vertical directions were important for detecting the pathological gait specific to iNPH. Therefore, we created two indices that combined the directional acceleration fluctuations, which were trunk acceleration index and forward × vertical acceleration fluctuation. In addition, the 95% CE volume for 3D plots of chronological changes of tri-axial accelerations which was defined in our previous study was also used to evaluate the probability of the pathological gait specific to iNPH. In our previous study, the severity of gait disturbance in iNPH was strongly associated with decreases in all directions of the trunk acceleration fluctuation, including lateral acceleration (Yamada et al., 2019). However, a pathological gait feature specific to iNPH was more specifically associated with decreases in the forward and vertical accelerations in trunk. Our findings are consistent with previous studies on gait analyses using a 3D optical motion capture system and/or force plates (Gard et al., 2004; Orendurff et al., 2004; Moe-Nilssen and Helbostad, 2005; Hernandez et al., 2009; Hurt et al., 2010; Arvin et al., 2016; Tesio and Rota, 2019). In an ideal gait model using the inverted pendulum motion, the gravitational potential energy due to the vertical rise of the center of mass is converted into propulsive kinetic energy in the forward direction (Gard et al., 2004; Orendurff et al., 2004). Additionally, the relevance of lateral movement with forward and vertical movements is considered to be clinically important in terms of a falling mechanism (Orendurff et al., 2004; Moe-Nilssen and Helbostad, 2005). As the walking speed increases, the body center of mass motion increases in the vertical direction and, consequently, decreases in the lateral direction (Orendurff et al., 2004; Moe-Nilssen and Helbostad, 2005). In general, compared to younger adults, older adults walk slowly with a shorter step length due to decreased forward and upward movements of the body center of mass, and they also adopt a wider step width due to increased movement in the lateral direction (Hernandez et al., 2009; Hurt et al., 2010; Arvin et al., 2016; Tesio and Rota, 2019). However, compared to healthy elderly individuals, frail elderly individuals are reported to exhibit less motion variability of the center of mass in the lateral direction, despite the maintenance of motion variabilities in forward and vertical directions (Moe-Nilssen and Helbostad, 2005).

Each pathological gait feature specific to iNPH, such as short-stepped gait, shuffling gait, and wide-based gait, could not be clearly distinguished using the directional trunk acceleration fluctuations, for any combination of parameters or with 95% CE volume during straight walking. The first reason for a lack of differentiation is that pathological gait features might be closely related to each other (Nutt et al., 1993). In theory, decreases in forward and vertical acceleration result in a slower walking speed with a smaller stride, which is characteristic of a short-stepped and shuffling gait. A slower walking speed also leads to instability and a wide-based gait due to the greater lateral sway of the body center of mass (Hernandez et al., 2009; Hurt et al., 2010; Arvin et al., 2016; Tesio and Rota, 2019). The second reason is that the functional localization for various pathological gait features specific to iNPH might be in complex locations at a higher level of expression (Lenfeldt et al., 2008; Ogata et al., 2017). Lenfeldt et al. reported that iNPH is a syndrome related to a reversible suppression of frontal periventricular cortico-basal ganglia-thalamo-cortical pathways (Lenfeldt et al., 2008). Recently, Ogata et al. demonstrated that functional connectivity related to gait disturbance in iNPH is concentrated in the frontal lobe (Ogata et al., 2017).

The greatest advantage of this research is that any researcher or clinician can easily and quantitatively evaluate pathological gait using an iPhone application. Instruments such as 3D optical motion capture systems or force plates are prohibitively expensive and may be unsuitable for universal clinical use. In comparison, our method can be applied easily without considerable amounts of time and money. Consequently, the novel trunk acceleration indices measured by an iPhone is useful for screening the pathological gait features specific to iNPH and can be applied in multicentre collaborative studies to assess the probability of an iNPH-specific pathological gait and changes after CSF tap-test or shunt surgery.

### Limitations

Some limitations to our study warrant discussion. First, the reliability and validity of the trunk acceleration fluctuations recorded by an iPhone have not been verified in this study. The gold standard for evaluating gait patterns is obtaining measurements using 3D optical motion capture systems and/or force plates, although the translation or acceleration of the body center of mass was reported to be closely related to locomotion of the lower limbs (Orendurff et al., 2004; Hurt et al., 2010; Tesio and Rota, 2019). Second, pathological gait features were assessed subjectively. In our previous study, gait assessment proved to be poorly consistent among raters (Ishikawa et al., 2019a). Therefore, we did not examine the association between the trunk acceleration indices and each pathological gait feature, instead examined the association with the pathological gait specific to iNPH, which was strongly associated with the severity of gait disturbance. We will conduct the next gait analysis by combining kinematic data from the 3D trunk acceleration and motion capture systems. Third, all gait features of 68 healthy elderlies were treated as normal, although their gait features were not assessed subjectively. Finally, we used a ratio of the simple sum of the eight pathological gait features specific to iNPH as an evaluation index for gait analysis. However, whether the gait features need to be weighted could not be verified.

## Conclusions

We demonstrate, for the first time, that pathological gait specific to iNPH can be assessed quantitatively by 3D trunk accelerations during simple, straight walking. Trunk acceleration recorded by a smartphone inertial accelerometer is useful for detecting pathological gait. As the trunk acceleration fluctuation reduces in the forward and vertical directions, the probability of a pathological gait specific to iNPH is high. In the future, 3D trunk acceleration fluctuation recorded by a wearable device or smartphone in a pocket may be a useful tool for detecting the probability of a pathological gait and fall risk.

## Data Availability Statement

The raw data supporting the conclusions of this article will be made available by the authors, without undue reservation.

## Ethics Statement

The studies involving human participants were reviewed and approved by the ethics committees for human research of Rakuwakai Otowa Hospital. The patients/participants provided their written informed consent to participate in this study.

## Author Contributions

SY made substantial contributions to the conception and design of the work, data acquisition, statistical analysis, and interpretation of the data. YA made substantial contributions to the development of the programming code for the data management system used for this study. MI substantially involved in the acquisition of data and the supervision of the study. MY and KY were also substantially involved in the acquisition of data. KN made substantial contributions to the critical revision of the manuscript for intellectual content and supervised the study. All authors contributed to the article and approved the submitted version.

## Conflict of Interest

YA was employed by company Digital Standard Co., Ltd., Osaka, Japan. The remaining authors declare that the research was conducted in the absence of any commercial or financial relationships that could be construed as a potential conflict of interest.
